# GLP-1 receptor agonists in eye disease: a comprehensive review of current research and future potential

**DOI:** 10.1186/s12886-025-04559-x

**Published:** 2026-01-08

**Authors:** Yu Luo, Yanting Xia, Xiaohong Gong, Meiling Hao, Qiping Wei, Liang Liao

**Affiliations:** 1 Dongfang Hospital, Beijing University of Chinese Medicine (The Second Clinical Medical College of Beijing University of Chinese Medicine), Beijing, 100078 China; 2 Beijing University of Chinese Medicine, Beijing, 100029 China

**Keywords:** GLP-1 receptor agonist, Diabetic retinopathy, Glaucoma, Age-related macular degeneration, Nonarteritic anterior ischemic optic neuropathy, Dry eye disease, Neuroprotection, Ophthalmology

## Abstract

**Objective:**

To synthesize the current preclinical and clinical evidence on the utility and potential mechanisms of glucagon-like peptide-1 receptor agonists (GLP-1RAs) as a therapeutic strategy for major ophthalmic diseases, including diabetic retinopathy, glaucoma, and age-related macular degeneration.

**Methods:**

A comprehensive literature search was conducted to synthesize the current preclinical and clinical evidence. Electronic databases including PubMed, Embase, and Web of Science were searched from inception until August 2025 using keywords such as “GLP-1 receptor agonist,” “diabetic retinopathy,” “glaucoma,” “age-related macular degeneration,” “ocular disease,” and “neuroprotection.” The search focused on identifying preclinical studies, large-scale retrospective cohort studies, and post-hoc analyses of major clinical trials that evaluated the utility and potential mechanisms of GLP-1RAs in ophthalmic diseases. Studies were screened for relevance based on title and abstract, and full texts were retrieved for detailed assessment.

**Results:**

Despite heterogeneity in study designs and populations, a largely consistent association was found between GLP-1RA use and a reduced incidence of glaucoma. For diabetic retinopathy, the evidence was nuanced, indicating potential long-term neurovascular protection but also a risk of transient early worsening. For age-related macular degeneration, findings were dichotomous, suggesting a protective effect against non-exudative forms but a potential increased risk for neovascular disease.

**Conclusion:**

GLP-1RAs show significant promise as a potential disease-modifying therapy for neurodegenerative and inflammatory eye diseases, acting through both systemic metabolic improvements and direct ocular mechanisms, representing a paradigm shift beyond their metabolic indications.

## Introduction

Glucagon-like peptide-1 (GLP-1), an incretin hormone secreted by intestinal enteroendocrine cells, is pivotal for postprandial glucose homeostasis through its modulation of insulin and glucagon secretion, appetite suppression, and gastric emptying [1]. The therapeutic utility of native GLP-1 is limited by its rapid degradation [2], primarily mediated by dipeptidyl peptidase-4 (DPP-4) and secondarily by neutral endopeptidases (NEPs) and renal clearance [3, 4]. GLP-1 Receptor Agonists (GLP-1RAs) are a class of pharmacotherapeutic agents that mimic the physiological actions of endogenous GLP-1 [5]. By exhibiting high binding affinity for GLP-1 receptors (GLP-1 Rs), they potentiate glucose-dependent insulin secretion from pancreatic β-cells [6]. Consequently, GLP-1RAs are recommended as a first-line injectable therapy for patients requiring pharmacological intervention to achieve glycemic control [7]. Beyond their primary application in the management of type 2 diabetes mellitus (T2DM), GLP-1RAs have demonstrated significant therapeutic potential in treating obesity. They facilitate weight reduction through multiple mechanisms, including delayed gastric emptying, suppressed appetite, and enhanced satiety signaling [8], with outcomes demonstrating substantial clinical efficacy [9, 10].

Although GLP-1RAs have revolutionized metabolic disease management, their potential therapeutic role in ocular diseases—particularly those driven by shared metabolic and inflammatory pathways—remains underexplored, despite growing preclinical evidence. Significant unmet clinical needs persist across major ophthalmic diseases. In the management of diabetic retinopathy (DR), anti-vascular endothelial growth factor (anti-VEGF) agents have emerged as first-line therapeutic interventions [11]. However, several critical challenges persist, including the substantial burden imposed by frequent intravitreal injections and a therapeutic focus that predominantly addresses vascular pathology while offering limited interventions for the underlying neurodegenerative processes. Glaucoma represents the leading global cause of irreversible blindness, with ongoing demographic shifts toward an aging population, its prevalence is projected to increase substantially in the coming decades [12]. Notably, current therapeutic interventions are incapable of restoring visual function lost to this neurodegenerative condition. Age-related macular degeneration (AMD) manifests a complex pathogenesis characterized by intricate interactions among oxidative stress, inflammatory responses, and metabolic dysregulation [13]. While anti-VEGF therapies have revolutionized the treatment of neovascular AMD (nAMD), a critical therapeutic gap persists. Specifically, there remains a complete absence of approved treatments for late-stage atrophic AMD; no therapeutic interventions have received regulatory approval to slow or halt the progression of geographic atrophy (GA) or to reverse the associated macular tissue degeneration [14].

While the conventional therapeutic applications of GLP-1RAs have primarily focused on metabolic disorders, their multifaceted neuroprotective properties and anti-inflammatory mechanisms present a distinctive therapeutic opportunity for ophthalmic pathologies, potentially offering direct ocular benefits beyond systemic metabolic control. This review will synthesize the current evidence, explore the potential mechanisms of action, and discuss the future prospects of utilizing GLP-1RAs as a novel treatment strategy for a range of debilitating eye diseases.

## Literature search strategy

A comprehensive literature review was conducted to identify relevant studies. Electronic databases, including PubMed, Embase, and the Web of Science, were searched from their inception until August 2025. Search terms included “GLP-1 receptor agonist,” “diabetic retinopathy,” “glaucoma,” “age-related macular degeneration,” “ocular disease,” and “neuroprotection.” The search included preclinical studies, large-scale retrospective cohort studies, and post-hoc analyses of major cardiovascular outcome trials (CVOTs). Studies were screened based on title and abstract for relevance. Inclusion criteria were: (1) original research evaluating GLP-1RAs in the context of ophthalmic disease, (2) published in English. Exclusion criteria were: (1) reviews, commentaries, or editorials; (2) case reports; (3) non-English publications. To enhance screening efficiency, duplicates were removed using EndNote 20. Two reviewers independently screened all identified records based on predefined inclusion and exclusion criteria. Discrepancies were resolved through discussion or adjudication by a third reviewer when consensus could not be reached.

**Fig. 1:**
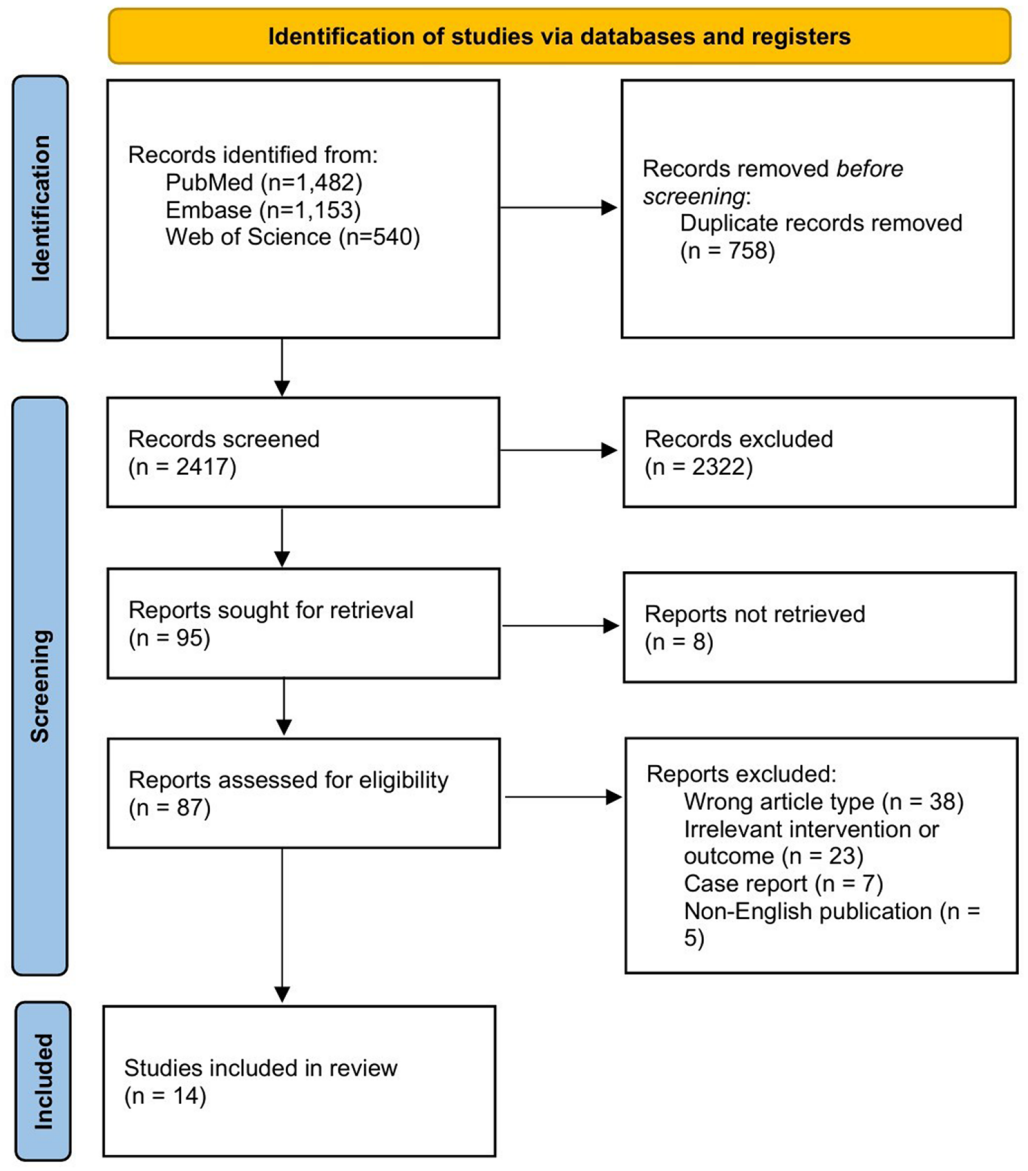
Flow diagram of the literature selection process

Data on study design, population, intervention, and key ophthalmic outcomes were extracted. We systematically extracted data on primary outcomes (visual acuity, retinal thickness, disease progression) and secondary outcomes (microvascular changes, inflammatory markers, adverse events), capturing all reported time points and analysis methods. All numerical data were double-checked against original publications. Study characteristics (participant demographics, intervention details, funding sources) were collected, with missing data addressed through author contact and conservative imputation. Figure 1 presents a flowchart of the study selection process.

## Biological characteristics of GLP-1RAs

### Molecular structure and pharmacological properties

Due to the suboptimal in vivo stability of the native GLP-1 peptide, GLP-1RAs were engineered with structural modifications that mimic endogenous GLP-1 while achieving a prolonged duration of action [15]. Representative GLP-1RAs are classified by their pharmacokinetic profiles: first-generation, short-acting formulations such as exenatide and lixisenatide are primarily utilized for postprandial glycemic control; second-generation, long-acting agents include liraglutide and semaglutide, with the latter demonstrating notable efficacy in weight reduction and neuroprotection. Other key agents in this class are dulaglutide and the novel dual GIP/GLP-1 receptor agonist, tirzepatide [16–19]. While united by a common mechanism, these agents vary significantly in their molecular structure, size, and half-life—properties that may influence their ability to penetrate the blood-retinal barrier (BRB) (Table 1). The structural diversity of these agents underpins their distinct pharmacological properties.

**Table 1:** Summary of key GLP-1 receptor agonists discussed in the review

Agent	Class	Administration Frequency	Half-life	Key Structural Feature	Known BRB/BBB Penetration	References
Exenatide	Exendin-4 Derivative	Once weekly (ER); Twice daily (immediate-release)	~2.4 hours (immediate-release)	Synthetic version of exendin-4 peptide; naturally resistant to DPP-4 degradation at its N-terminus. ER version uses microsphere technology.	Demonstrated to cross the BBB in animal models; considered relatively small molecule (~4.2 kDa).	[20–22]
Lixisenatide	Exendin-4 Derivative	Once daily	~3 hours	Exendin-4 analog with a C-terminal modification (addition of six lysine residues) that slightly prolongs its action.	Demonstrated to cross the BBB in animal models.	[20, 21]
Liraglutide	Human GLP-1 Analog	Once daily	~13 hours	Acylation with a C16 fatty acid chain, which enables non-covalent binding to serum albumin, protecting it from degradation and renal clearance.	Well-documented to cross the BBB and exert central neuroprotective effects in multiple preclinical models.	[21, 23]
Semaglutide	Human GLP-1 Analog	Once weekly (subcutaneous); Once daily (oral)	~165 hours	Enhanced albumin binding via a more complex C18 fatty diacid side chain and linker; amino acid substitution provides stronger resistance to DPP-4.	Believed to cross the BBB, enabling central appetite suppression. Penetration extent is under investigation but central effects are evident.	[24]
Dulaglutide	Human GLP-1 Analog (Fusion Protein)	Once weekly	~5 days	Two GLP-1 analog molecules are covalently fused to a modified human IgG4 Fc fragment, creating a large molecule that resists renal clearance.	Large molecular size (~63 kDa) is expected to significantly limit direct BBB/BRB penetration. Effects may be mediated indirectly or via circumventricular organs.	[25]
Tirzepatide	Dual GIP/GLP-1 Receptor Co-agonist	Once weekly	~5 days	A single 39-amino acid peptide engineered for dual agonism at both GIP and GLP-1 receptors. A C20 fatty diacid moiety allows for strong albumin binding.	Expected to cross the BBB based on its structure and clinically observed central effects (e.g., on appetite and weight reduction).	[16, 26–28]

The structural diversity of these agents underpins their distinct pharmacological properties. Both exenatide and lixisenatide, derivatives of exendin-4, feature a modification at the second N-terminal amino acid to confer resistance to DPP-4-mediated degradation [20]. Liraglutide, an acylated human GLP-1RA produced via recombinant DNA technology, exhibits 97% sequence homology with endogenous human GLP-1(7–37). Following subcutaneous administration, liraglutide reaches peak plasma concentrations within 8–12 hours and has an elimination half-life of approximately 13 hours [23]. Semaglutide shares 94% sequence homology with human GLP-1, and its prolonged action is attributed to strong albumin binding and resistance to DPP-4 degradation [24]. Dulaglutide is a fusion protein consisting of two identical disulfide-linked chains, each composed of a GLP-1 analog (90% homology with native GLP-1) covalently linked to a modified human IgG4 Fc domain [25]. Tirzepatide represents a new class of single-molecule dual agonists for both GIP and GLP-1 Rs, developed from pioneering research on multi-receptor peptides [26]. It is a 39-amino acid linear peptide structurally modified for an extended plasma half-life [27]. While tirzepatide has a comparable binding affinity for the GIP receptor, its affinity for the GLP-1 R is approximately fivefold weaker than that of native GLP-1. Notably, it acts as a biased agonist at the GLP-1 R, preferentially stimulating cyclic adenosine monophosphate (cAMP) production [28].

While the metabolic efficacy of GLP-1RAs is extensively validated, their potential in neuro-ophthalmology is contingent upon their ability to penetrate the blood-brain barrier (BBB) and the BRB. The neuroprotective potential of GLP-1 has been established in models of neurodegenerative disorders, including Parkinson’s disease and Alzheimer’s disease (AD), with evidence suggesting it can modify disease progression and ameliorate pathology [29–32]. This therapeutic effect is predicated on the ability of GLP-1RAs to cross the BBB and exert direct physiological effects within the brain [21, 22]. Although much of this evidence derives from animal models, making direct extrapolation to humans complex, significant functional commonalities exist between the murine and human BBB, including glucose transport, the presence of circumventricular organs (CVOs), and saturable transport systems [33]. Whereas central nervous system (CNS) effects are determined by BBB penetration, ocular efficacy is governed by the traversal of the BRB. Analogous to the BBB, the BRB has an inner component (iBRB) of retinal endothelial cells and an outer component (oBRB) formed by tight junctions between retinal pigment epithelial (RPE) cells [34]. These barriers can exhibit differential permeability to pharmaceutical compounds. Therefore, a pivotal question is whether the demonstrated BBB permeability of certain GLP-1RAs translates to effective penetration of the BRB to achieve therapeutic concentrations in retinal tissues.

### Expression of GLP-1 receptors in the ocular region

While the pharmacokinetic properties of GLP-1RAs determine their systemic availability, their therapeutic potential in ocular diseases is fundamentally contingent upon the presence and localization of GLP-1 R in target tissues. The GLP-1 R is a member of the class B family of G protein-coupled receptors (GPCRs) [35], and investigations have confirmed its expression within the human retina [36]. The localization of this receptor is highly specific; current evidence indicates that GLP-1 R expression is found predominantly within the ganglion cell layer (GCL), exclusively detected in a discrete subpopulation of neurons [37]. Morphologically, these GLP-1 R-positive cells display characteristic neuronal phenotypes, such as spherical nuclei and horizontal projections.

This specific localization within the GCL is of profound clinical relevance, as retinal ganglion cells are the primary neurons that degenerate in glaucoma and are also critically affected during the early neurodegenerative stages of diabetic retinopathy [38, 39]. However, it is equally notable that GLP-1 R expression is reportedly undetectable in retinal and choroidal vasculature, the RPE, or other ocular structures [37]. This finding suggests that the primary mechanism of GLP-1RAs in the retina is likely direct neuroprotection. It also implies that any beneficial effects on vascular or RPE pathology may be mediated indirectly, either through neuronal signaling pathways within the retina or via systemic anti-inflammatory and metabolic improvements.

## Mechanisms and potential targets of GLP-1RAs

### Neuroprotective effects

The established anti-inflammatory and neuroprotective effects of GLP-1RAs within the central nervous system suggest their therapeutic potential across a spectrum of neurodegenerative disorders [40, 41]. In preclinical models of glaucoma and DR, GLP-1RAs have demonstrated robust neuroprotective effects in ocular tissues, primarily by modulating oxidative stress, inflammation, mitochondrial function, and synaptic plasticity [42, 43].

In preclinical models of glaucoma, neuroprotection appears to be strongly linked to the anti-inflammatory properties of GLP-1RAs. For instance, NLY01, a long-acting PEGylated GLP-1RA with an extended half-life [41], effectively mitigates retinal ganglion cell (RGC) apoptosis induced by elevated intraocular pressure, a process mediated by a well-defined inflammatory cascade [44]. Ocular hypertension triggers the production of pro-inflammatory cytokines by immune cells, which in turn drives the transformation of neuroprotective astrocytes into a neurotoxic A1-reactive phenotype, ultimately leading to RGC degeneration [45]. NLY01 exerts its neuroprotective effect by potently suppressing this pathogenic inflammatory axis, thereby attenuating the formation of cytotoxic A1 astrocytes and preserving RGC viability [42]. Corroborating these findings, other GLP-1RAs have similarly shown neuroprotective properties in murine models of ocular hypertensive glaucoma, manifesting as enhanced RGC survival, reduced microglial/macrophage activation, and amelioration of optic nerve axon degeneration [46].

In the context of diabetic retinopathy, Müller cells, which constitute approximately 90% of retinal glia and are critical for neuronal support [47], emerge as a key therapeutic target. Under diabetic conditions, these cells undergo reactive gliosis, contributing to the inflammatory environment [48]. Exendin-4 (E4), a long-acting GLP-1RA, has demonstrated neuroprotective effects in early experimental DR [49, 50]. In the Goto-Kakizaki (GK) rat, a spontaneous model of T2DM [51], intravitreal E4 prevented cell loss in the GCL and outer nuclear layer (ONL) without affecting systemic glucose levels, suggesting a local retinal mechanism. At the cellular level, E4 directly targets Müller cells to attenuate reactive gliosis, preserving the critical Bcl-2/Bax and Bcl-xL/Bax pro-survival ratios and consequently mitigating mitochondrial-mediated apoptosis via reduced caspase-3 activation [52].

Further studies in DR models have elucidated additional protective pathways. Lixisenatide inhibits neuroinflammation by promoting the proteasomal degradation of thioredoxin-interacting protein (TXNIP) in ocular tissues via the PI3K/Akt signaling pathway, an effect more potent than that of insulin. This intervention significantly attenuates retinal cell apoptosis and ameliorates pathological thickening of the inner nuclear layer (INL) and nerve fiber layer (NFL) [53]. Similarly, in a diabetic rat model induced by a high-fat diet and streptozotocin (STZ) injection [54, 55], liraglutide was shown to mitigate the hyperglycemia-induced decline in RGC-5 cell viability and improve the structural integrity and organization of the GCL [56].

Mechanistically, these neuroprotective effects are underpinned by the activation of canonical GLP-1 R signaling pathways within the retina. In the retinas of diabetic mice treated with GLP-1RAs, a significant elevation in both cAMP and pAKT levels was observed, indicating successful target engagement and triggering of downstream pro-survival signals [36]. Furthermore, GLP-1RAs can counteract the diabetes-induced downregulation of the glutamate-aspartate transporter (GLAST), a key glial transporter responsible for clearing extracellular glutamate [57]. By restoring GLAST expression, GLP-1RAs may protect retinal neurons from excitotoxicity and subsequent apoptosis [36]. Collectively, these preclinical studies illustrate that GLP-1RAs protect retinal neurons through a multi-pronged approach involving anti-inflammatory, anti-apoptotic, and anti-excitotoxic mechanisms.

### Vascular protection and anti-inflammatory effects

Emerging evidence suggests that GLP-1RAs hold considerable therapeutic promise in ophthalmic pathologies, particularly through their vasoprotective and anti-inflammatory mechanisms. This pharmacological effect is highly relevant for addressing the fundamental pathogenic processes of DR. While long understood as a microvascular disease, the central role of neuroinflammation and immune dysregulation is now increasingly clear. Recent multi-omics analyses by Cappellani et al., for instance, have provided strong genetic and proteomic evidence linking DR pathogenesis directly to the activation of specific immune pathways, including the complement system and adaptive T-cell responses [53, 58, 59]. This refined understanding of DR as an immunoinflammatory disease provides a strong rationale for exploring immunomodulatory agents like GLP-1RAs. GLP-1RAs appear to exert dual therapeutic benefits by concurrently ameliorating neuronal and vascular pathologies through stabilization of the BRB and suppression of inflammation-driven angiogenesis.

The vasomodulatory effects of GLP-1RAs also extend to glaucoma models, although the observed outcomes differ. Systemic administration of semaglutide conferred discernible vasoprotective and anti-neuroinflammatory benefits in a rodent model of glaucoma, primarily manifesting as attenuated intraocular pressure elevation and suppression of pathological astrocyte activation and vascular remodeling. Intriguingly, however, semaglutide treatment did not directly improve retinal ganglion cell survival in this context. Instead, the pharmacological intervention significantly enhanced the structural complexity (fractal dimension and lacunarity) of astrocytic networks and promoted a more organized retinal vasculature. These morphological improvements suggest a potential mechanism for mitigating vascular dropout and capillary degeneration in glaucomatous conditions, even if direct, short-term neuroprotection was not observed [60].

The vasoprotective effects are most evident in models of diabetic retinopathy, where a hallmark of the disease is the breakdown of the BRB and subsequent retinal vascular leakage [61]. In GK rats, E4 treatment significantly suppressed Evans blue leakage from retinal vessels. This was achieved by activating retinal GLP-1 R and inhibiting the overexpression of placental growth factor (PLGF) and intercellular adhesion molecule-1 (ICAM-1), thereby stabilizing tight junction proteins and preserving BRB integrity. Mechanistically, E4‘s primary effect was to alleviate inflammatory responses and vascular abnormalities, with relatively modest inhibitory effects on VEGF [62]. Consistent with these findings, locally administered GLP-1RAs in db/db mice also prevented diabetes-induced albumin leakage and suppressed the overexpression of VEGF and IL-1β, thus preserving BRB integrity [36]. These studizes collectively suggest that GLP-1RA treatment not only mitigates neurodegeneration but also ameliorates early-stage microvascular damage.

### Metabolic regulatory functions

While the vasomodulatory and anti-inflammatory effects of GLP-1RAs target downstream pathological events, their fundamental therapeutic potential may lie in the direct modulation of metabolic dysregulation. Ocular tissues exhibit exceptionally high energy demands: photoreceptors require substantial ATP for phototransduction; the RPE engages in active transport to maintain the BRB; and high-frequency synaptic transmission imposes further metabolic burdens [63]. This high metabolic demand renders ocular tissues particularly vulnerable to metabolic dysregulation, which can precipitate an energy crisis leading to retinopathy, AMD, and other pathologies. This metabolic vulnerability, however, also presents a prime therapeutic target for metabolic modulators such as GLP-1RAs.

In AMD, this metabolic link is evident. Drusen, the pathological hallmark of AMD, are lipid-rich extracellular deposits from the RPE that impair cellular function and serve as a nidus for chronic inflammation [64]. Elevated free fatty acids (FFAs) can drive the intracellular accumulation of lipid droplets (LDs) within RPE cells, inducing oxidative stress and epithelial-mesenchymal transition (EMT)—processes strongly associated with AMD progression [65, 66]. Liraglutide has been shown to ameliorate this lipid droplet accumulation in ARPE-19 cells by activating the AMP-activated protein kinase (AMPK) pathway, which in turn attenuates EMT, enhances lipophagy, and mitigates oxidative stress [67].

Beyond lipid metabolism, GLP-1RAs exert profound effects on mitochondrial homeostasis in retinal neurons. Research has confirmed that RGC-5 cells express GLP-1 R and are susceptible to high-glucose-induced mitochondrial damage [43]. Under hyperglycemic conditions, RGCs exhibit significant mitochondrial impairment, including cristae dissolution, swelling, vacuolar degeneration, elevated reactive oxygen species (ROS) production, and diminished mitochondrial membrane potential (MMP) [68, 69]. GLP-1RAs appear to counteract this damage at multiple levels. Exenatide treatment ameliorates these morphological alterations and inhibits the mitochondrial apoptotic pathway by suppressing cytochrome c release and favorably modulating the expression of pro-apoptotic (Bax, caspase-3) and anti-apoptotic (Bcl-2) proteins, thereby protecting RGC-5 cells from hyperglycemic injury [43].

In addition to regulating apoptosis, GLP-1RAs also fine-tune mitochondrial quality control via mitophagy. Mitochondria are highly dynamic organelles pivotal for ATP production, metabolism, and ROS signaling [70]. Liraglutide administration significantly mitigates excessive high glucose-induced mitophagy, as evidenced by reduced autophagosome formation and a normalization of LC3A/B and p62 expression. In energy-demanding RGCs, while basal mitophagy is physiologically essential, excessive activation can lead to energy depletion and cellular demise. By restoring this crucial balance, liraglutide helps preserve mitochondrial integrity and neuronal viability [56].

### Anti-inflammatory

Beyond their established metabolic functions, GLP-1RAs exert direct immunomodulatory effects within the central nervous system. A key mechanism involves their interaction with glial cells, as recent studies show that activated microglia and astrocytes can induce the expression of GLP-1 R, making them direct targets for GLP-1 mimetics [41]. For instance, GLP-1 treatment attenuates the endotoxin-induced release of the potent proinflammatory cytokine IL-1β [71], which is known to inhibit neuronal transmission and promote apoptosis [72]. In preclinical models of AD, this translates to significant therapeutic effects. Liraglutide treatment reduced the population of activated microglia by 50% in APP/PS1 mice [73]. Mechanistically, GLP-1RAs suppress neuroinflammation by shifting microglia from a pro-inflammatory to a phagocytic, anti-inflammatory phenotype. They reduce the release of cytokines like IL-1β, IL-6, and TNF, upregulate the anti-inflammatory cytokine TGF-β, and downregulate the expression of disease-associated microglial (DAM) genes, ultimately enhancing Aβ clearance [74]. This demonstrates a profound capacity to quell neuroinflammation.

This potent anti-inflammatory capacity also translates to pathologies of the ocular surface, such as diabetic dry eye disease. Immunohistochemical analysis has revealed GLP-1 R expression in the acinar and ductal epithelium of the lacrimal gland, although this expression is significantly downregulated in diabetic models. Topical administration of a liraglutide-containing ophthalmic solution reversed this pathology, markedly increasing tear fluid secretion and attenuating diabetes-induced inflammation in the lacrimal gland [75]. Similarly, SM102, a novel long-acting GLP-1RA structurally related to semaglutide, demonstrated robust efficacy in the db/db mouse model. SM102 treatment enhanced tear secretion, prolonged tear film breakup time, reversed corneal tissue damage, and increased conjunctival goblet cell density. These benefits were associated with a rebalancing of the cytokine milieu—reducing pro-inflammatory IL-6, IL-1β, and TNF-α while elevating anti-inflammatory TGF-β1—mediated by the suppression of the MAPK signaling pathway [76]. These findings highlight the therapeutic versatility of GLP-1RAs, addressing inflammatory conditions from the CNS to the anterior segment of the eye.

The significance of these immunomodulatory effects is further underscored by recent advances in understanding DR pathogenesis. As highlighted by Cappellani et al., the molecular landscape of DR is deeply intertwined with the dysregulation of the adaptive immune system and the complement cascade [59]. The established ability of GLP-1RAs to suppress glial cell activation and modulate cytokine release—key drivers of these very pathways—positions them as a uniquely targeted therapeutic strategy. This offers a compelling mechanistic rationale for their use in DR, suggesting they do not merely offer a generic anti-inflammatory benefit, but may directly intervene in the specific immunological events now known to perpetuate retinal damage.

**Fig. 2:**
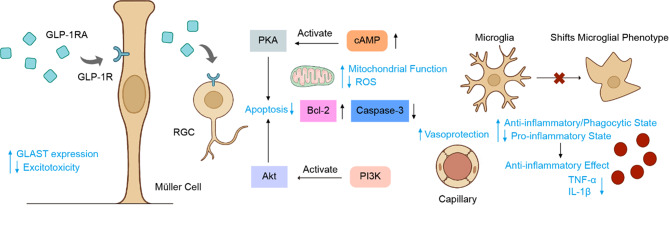
Proposed multifaceted mechanisms of GLP-1RAs in the retinal neurovascular unit. Activation of the GLP-1 R on key retinal cells such as RGCs and müller cells initiates two primary downstream signaling cascades. The PI3K/Akt pathway promotes cell survival by suppressing apoptosis (e.g., upregulating bcl-2 and downregulating caspase-3). The PKA/cAMP pathway enhances mitochondrial function, reduces oxidative stress, and contributes to neuroprotection. Together, these pathways orchestrate a coordinated protective response: (1) neuroprotection, through direct anti-apoptotic effects on RGCs and reduced excitotoxicity via enhanced GLAST expression in müller cells; (2) anti-inflammation, by shifting microglia from a pro-inflammatory to a protective phenotype, thereby reducing cytokine release (TNF-α, IL-1β); and (3) Vasoprotection, by enhancing the integrity of the blood-retinal barrier in capillaries

Figure 2 provides a schematic representation of how GLP-1 R activation on key retinal cells orchestrates a coordinated protective response, integrating neuroprotective, anti-inflammatory, and vasoprotective actions through convergent signaling pathways.

## Progress in clinical research

### Diabetic retinopathy

As a leading cause of vision loss in working-age adults, DR presents a major therapeutic challenge [61, 77]. Given the established neuroinflammatory and vasomodulatory mechanisms of GLP-1RAs, their potential to modify DR progression has become a topic of intense clinical investigation and debate. Indeed, early retrospective cohort studies and post-hoc analyses suggested that GLP-1RAs might delay DR progression or improve outcomes, with one study reporting that 80% of patients on continuous therapy achieved either improvement or stabilization of their DR status [78, 79]. However, the clinical picture is far from straightforward. A recent retrospective cohort analysis revealed that, compared to the combination of SGLT-2 inhibitors with insulin, the concomitant use of GLP-1RAs and insulin was associated with a significantly elevated risk of DR and diabetic macular edema [80].

This complexity is further echoed in analyses that differentiate between disease stages. While GLP-1RAs have been linked to a statistically significant elevation in the risk of early-stage DR events compared to placebo, they may conversely demonstrate a protective effect against the progression to advanced-stage diabetic retinopathy when compared to insulin therapy [81]. The concern regarding an early increase in risk was most prominently highlighted by the SUSTAIN-6 trial, which found that patients undergoing semaglutide treatment exhibited an elevated risk of diabetic retinopathy complications [82]. This apparent paradox—an early risk coupled with a potential long-term benefit—demanded a mechanistic explanation.

The leading hypothesis to reconcile these findings is the “early worsening” phenomenon, where rapid and substantial glycemic improvement temporarily exacerbates pre-existing DR. This has been a long-recognized event in patients with type 1 diabetes [83, 84]. To probe whether semaglutide exerts a direct adverse vascular effect, a separate randomized controlled trial specifically investigated its impact on retinal physiology. The trial failed to demonstrate any statistically significant impact of semaglutide on retinal oxygenation or vascular diameter, arguing against a direct vasotoxic mechanism. However, semaglutide administration was associated with a marginal increase in central retinal thickness; importantly, this effect was attenuated after adjusting for glycated hemoglobin (HbA1c) levels, suggesting a strong correlation with the degree of glycemic improvement [85]. This concept is powerfully substantiated by a comprehensive meta-analysis encompassing seven major CVOTs, which concluded that the elevated risk of DR progression associated with semaglutide was driven by the magnitude and rapidity of HbA1c reduction, rather than a direct drug effect [86].

The underlying pathophysiology of this early worsening is believed to involve hemodynamic shifts in a retinal microvasculature that has lost its capacity for autoregulation. The retinal capillary damage central to DR leads to tissue hypoxia, altered venous oxygen saturation, and a compromised ability to regulate blood flow [87–89]. These changes are not uniform, with the macular area typically exhibiting dilation while the periphery suffers from ischemic hypoxia [90, 91]. A rapid metabolic correction can overwhelm this fragile, dysregulated system, potentially worsening vascular leakage and macular edema.

Despite the coherence of the early worsening hypothesis, the narrative is further complicated by a large body of real-world evidence that does not reflect the risk signals seen in certain trials. Multiple large-scale analyses of observational data, including those comparing GLP-1RAs to SGLT-2 inhibitors and other oral agents, have found no statistically significant association between GLP-1RA use and the exacerbation of DR or the development of sight-threatening retinopathy [92–95]. This discrepancy between controlled trials and real-world evidence highlights the complexities of translating trial findings to diverse patient populations.

In conclusion, the role of GLP-1RAs in the management of DR remains a nuanced and evolving topic. The potent glycemic-lowering capacity of some agents can induce a temporary worsening of DR, a phenomenon driven by rapid metabolic correction rather than direct toxicity. Yet, this risk must be balanced against a largely neutral safety profile in broad observational studies and a potential for long-term benefit. Definitive answers are eagerly awaited from ongoing trials like the FOCUS study (NCT03811561), which is specifically designed to clarify the long-term impact of semaglutide on diabetic eye disease [96].

### Glaucoma

Glaucoma is a leading cause of irreversible blindness characterized by progressive RGC degeneration [97]. Mounting evidence implicates chronic neuroinflammation and glial cell dysregulation as central drivers of this neurodegenerative process [98–100].

Given this neuroinflammatory underpinning, the established immunomodulatory and neuroprotective properties of GLP-1RAs make them a compelling therapeutic candidate for glaucoma. Preclinical evidence provides a potential mechanism, with animal models showing that GLP-1RAs can reduce intraocular pressure by inhibiting sodium-potassium ATPase and enhancing nitric oxide synthesis [101]. This hypothesis is strongly supported by a consistent body of evidence from large-scale retrospective clinical studies. Across diverse patient populations, GLP-1RA use has been associated with a reduced risk of glaucoma. In obese patients without diabetes, their use was linked to a significantly lower incidence and progression of primary open-angle glaucoma (POAG) and ocular hypertension compared to other weight-loss drugs [102]. Furthermore, studies have shown GLP-1RAs are associated with a modest decrease in intraocular pressure, particularly in individuals with elevated baseline intraocular pressure (IOP) [103], and a reduced risk of developing glaucoma in patients with T2DM [104]. This protective association has been repeatedly observed, linking GLP-1RA use to lower risks of POAG, ocular hypertension, and the need for first-line glaucoma treatment in large cohorts of T2DM patients [105–107]. A systematic meta-analysis has corroborated these findings, confirming a significant association between GLP-1RA utilization and reduced glaucoma incidence in retrospective studies [108]. While this body of evidence is highly encouraging, it is important to note that these findings are derived from studies with considerable methodological heterogeneity, a limitation that will be addressed in the Discussion. Consequently, prospective randomized controlled trials (RCTs) are imperative to establish definitive causality. Future directions should prioritize RCTs targeting early-stage glaucoma, with primary endpoints encompassing both structural (e.g., OCT-based RGC metrics) and functional (e.g., visual field preservation) outcomes, to validate the potential of GLP-1RAs as a true disease-modifying strategy in glaucoma management.

### Age-related macular degeneration

As a leading cause of central vision loss in the elderly, AMD is a multifactorial disease driven by chronic oxidative stress, inflammation, and metabolic dysregulation [109, 110].

Intriguingly, the current clinical evidence regarding GLP-1RAs in AMD is profoundly dichotomous, with their effect appearing to depend entirely on the disease subtype. On one hand, a retrospective cohort study demonstrated a significant protective association, linking GLP-1RA use to a reduced risk of nonexudative AMD, with an efficacy reported to be superior to that of metformin, insulin, and statins [111]. This benefit is putatively driven by the systemic amelioration of metabolic risk factors like high body mass index (BMI) and hyperlipidemia, though direct preclinical evidence remains lacking. However, this potential protective mechanism is overshadowed by alarming findings in nAMD. Another large-scale cohort study demonstrated that long-term GLP-1RA use was associated with a more than twofold, exposure-dependent increase in the risk of developing nAMD. The proposed mechanism for this adverse outcome involves a paradoxical effect where GLP-1RA-induced metabolic shifts cause transient retinal hypoxia, triggering the upregulation of pro-angiogenic chemokines like CXCL12 [112].

### Other ocular diseases

#### Ischemic optic neuropathy

Nonarteritic anterior ischemic optic neuropathy (NAION), the most prevalent form of acute optic neuropathy in the elderly, results from ischemic damage to the anterior optic nerve head due to small vessel hypoperfusion [113]. Recently, an emerging safety signal has linked potent GLP-1RAs like semaglutide and tirzepatide to NAION. Initial concerns were raised by an early, methodologically critiqued study and a series of case reports in which the majority of severe ocular complications presented as NAION [114, 115]. However, this anecdotal evidence is insufficient to establish causality. Subsequent, more rigorous retrospective analyses have yielded conflicting and nuanced results. One study found no significant increase in NAION risk with semaglutide compared to other agents when using broad diagnostic criteria; a marginally elevated risk was only observed with highly specific criteria and only in comparison to empagliflozin [116]. In contrast, a global pharmacovigilance study did report a significant and disproportionate association between semaglutide and reports of ischemic optic neuropathy (including NAION) [117]. Taken together, while NAION remains a rare event with low absolute risk, these findings constitute a signal that warrants heightened clinical vigilance, particularly in high-risk patients. Further mechanistic and prospective studies are required to establish a definitive causal relationship.

#### Dry eye disease

Dry Eye Disease (DED) is a multifactorial condition of the ocular surface characterized by a loss of tear film homeostasis, with a particularly high prevalence among individuals with diabetes (Diabetic Dry Eye Syndrome, DDES) [118–121]. The pathophysiology of DDES is complex, involving lacrimal gland mitochondrial dysfunction, neuro-inflammation, ocular surface dysbiosis, and meibomian gland dropout [122–124]. Consistent with their broad anti-inflammatory and metabolic regulatory functions discussed previously, GLP-1RAs have shown protective efficacy against DDES in empirical studies [125, 126]. However, the therapeutic landscape is nuanced. A comparative study suggested that patients with T2DM treated with SGLT2 inhibitors may have an even lower risk of developing DED than those on GLP-1RAs [127]. This finding highlights that while GLP-1RAs represent a promising therapeutic avenue for DDES, further comparative effectiveness research is needed to determine their relative efficacy within the modern antidiabetic armamentarium.

The clinical picture for GLP-1RAs in ophthalmology is nuanced, with evidence varying significantly across different diseases and study designs. Table 2 provides a comprehensive overview of the current clinical landscape, highlighting the divergent outcomes in conditions such as DR and AMD.

**Table 2:** Summary of clinical evidence for GLP-1 receptor agonists in major ophthalmic diseases

Ophthalmic Disease		Key Studies	Study Design	Main Findings	Key Limitations	Evidence Strength
Diabetic Retinopathy (DR)		SUSTAIN-6 (Marso et al. 2016) Meta-analysis of CVOTs (Albert et al. 2023) Various real-world cohort studies (e.g., Douros et al. 2018)	Post-hoc analysis of RCTs; meta-analysis; retrospective cohort studies	·RCTs (post-hoc): Show an increased risk of early DR complications, strongly associated with the magnitude and rapidity of HbA1c reduction. ·Real-world evidence: Multiple large cohort studies find no significant association with DR progression or exacerbation.	·RCT data: Derived from post-hoc analyses not designed for ophthalmic endpoints, posing a risk of detection bias and using non-standardized assessments. ·Retrospective studies: High risk of confounding by indication and selection bias.	Medium to High
Glaucoma		Niazi et al. 2024 Vasu et al. 2025 Sterling et al. 2023 Amaral et al. 2025	Large-scale retrospective cohort studies; systematic review and meta-analysis	·Strong and consistent association with a reduced incidence of POAG and ocular hypertension. ·Protective effect observed across diverse populations (with and without diabetes). ·Some studies report a modest reduction in IOP.	·Evidence is exclusively from retrospective designs, which cannot establish causality. ·Susceptible to confounding from healthy user effects (e.g., healthier lifestyle, better healthcare access).	Medium
Age-Related Macular Degeneration (AMD)	Non-exudative	Allan et al. 2025	Retrospective cohort study	·Linked to a significantly reduced risk of developing non-exudative AMD. ·Reported efficacy was superior to metformin or statins.	·Findings are based on a single large retrospective study and require independent validation. ·The proposed mechanism (systemic metabolic improvement) is not yet directly proven.	Low to Medium
	Neovascular	Shor et al. 2025	Large-scale retrospective cohort study	·Long-term, exposure-dependent use was associated with a more than 2-fold increased risk of developing neovascular AMD.	·This significant safety signal originates from a single retrospective source. ·Requires urgent validation in other datasets and prospective studies.	Low to Medium
Nonarteritic Anterior Ischemic Optic Neuropathy (NAION)		Hathaway et al. 2024 Lakhani et al. 2025 Cai et al. 2025	Case series; pharmacovigilance database analyses; retrospective cohort studies	·Evidence is mixed and conflicting. ·Pharmacovigilance data show a disproportionate reporting association. ·Some retrospective analyses find a marginally elevated risk with specific criteria, while others find no association.	·Evidence is primarily from sources that cannot establish causality (case reports, reporting bias). ·A definitive causal link has not been established, but the signal warrants clinical vigilance.	Low
Dry Eye Disease (DED)		Su et al. 2022 Fan et al. 2023	Population-based retrospective cohort studies	·Associated with a reduced risk of DED in patients with T2DM. ·However, one comparative study suggested that SGLT2 inhibitors might offer greater protection.	·Evidence is retrospective and cannot establish causality. ·Comparative effectiveness data is limited, highlighting the need for further research to clarify relative benefits.	Medium

^*^Evidence Strength Grading: Medium to High indicates evidence from post-hoc analyses of major RCTs combined with large-scale retrospective studies, though findings may show some nuances (e.g., early worsening vs. long-term benefit). Medium indicates a consistent body of evidence from multiple large-scale retrospective cohort studies or systematic reviews of such studies. Low to Medium indicates evidence primarily from a single large retrospective study or conflicting findings across multiple smaller studies, requiring independent validation. Low indicates evidence derived from sources that cannot establish causality, such as pharmacovigilance databases, case series, or preliminary/conflicting retrospective analyses

## Discussion

While GLP-1RAs hold considerable promise for ophthalmic disorders, particularly DR and glaucoma, significant hurdles remain in translating preclinical findings into routine clinical practice. To realize their full therapeutic potential, future research must rigorously address several critical dimensions, which collectively form a roadmap for the field.

The precise mechanisms underlying the ocular benefits of GLP-1RAs remain incompletely elucidated. Their effects likely arise from a convergence of pathways: indirectly, through systemic improvements in glycemic control and metabolism, and directly, via the activation of intraocular GLP-1Rs on cells like retinal Müller and ganglion cells, which triggers local anti-inflammatory, anti-oxidant, and anti-apoptotic responses. Disentangling the relative contributions of these pathways is a major challenge. Future studies employing sophisticated designs, such as ocular-specific GLP-1 R knockout models, are essential to determine if direct neuroprotective effects can occur independently of systemic metabolic correction.

All currently approved GLP-1RAs are administered systemically. For these agents to act on posterior segment tissues, they must traverse the formidable BRB, which significantly restricts drug penetration. Achieving therapeutic concentrations in the retina via systemic routes may require doses high enough to increase the risk of extraocular adverse effects [128]. Therefore, the development of novel drug delivery systems—whether by enhancing BRB penetration or through direct ocular targeting (e.g., long-acting intravitreal formulations)—is a critical and rate-limiting step for the clinical translation of these agents.

GLP-1RAs are not a monolithic entity but a diverse class of drugs with distinct structures and pharmacokinetic profiles. This class includes human GLP-1 analogs (e.g., liraglutide, semaglutide, dulaglutide) and E4 based therapies (e.g., exenatide) [129]. These agents vary in receptor affinity, duration of action, molecular size, and ability to cross biological barriers, which likely translates to different efficacy profiles in the eye. A crucial knowledge gap is the lack of head-to-head comparative studies evaluating their ocular effects. Systematic investigation is needed to identify which agents, if any, are best suited for ophthalmic applications, paving the way for a precision medicine approach.

The majority of preclinical evidence is derived from rodent models that often represent acute or subacute disease states. These models may not fully recapitulate the chronic, multifactorial nature of human ocular diseases like DR, which involves decades of metabolic dysregulation and low-grade inflammation [130–133]. The profound anatomical and pathophysiological disparities between these models and human disease create a significant “translational gap.” Acknowledging these limitations is key to interpreting preclinical data cautiously and designing more relevant experimental paradigms.

A significant limitation of the current clinical evidence is its reliance on post-hoc analyses of cardiovascular outcome trials and large-scale retrospective studies. The former were not designed with primary ophthalmic endpoints, leading to potential detection bias and non-standardized ocular assessments. The latter, while providing valuable real-world signals, are inherently susceptible to confounding by indication and selection bias, as patient characteristics and co-morbidities may differ significantly between treatment groups. These methodological limitations temper the strength of the conclusions and preclude a quantitative synthesis, highlighting the urgent need for prospective, dedicated ophthalmic RCTs.

When considering the clinical application of GLP-1RAs for ocular protection, a comparison with other modern antidiabetic agents, particularly SGLT2 inhibitors, is crucial. The evidence landscape for these two classes presents notable distinctions. For diabetic retinopathy, GLP-1RAs are associated with the ‘early worsening’ phenomenon linked to rapid glycemic control, a signal not typically observed with SGLT2 inhibitors, which generally show a neutral or modestly protective effect. Conversely, for DDES, some comparative evidence suggests that SGLT2 inhibitors may offer a greater risk reduction than GLP-1RAs [127]. These differences suggest that the choice of agent may depend on the specific ocular comorbidity and the patient’s baseline retinopathy status. Future head-to-head trials are needed to clarify the relative ocular benefits and risks of these important drug classes.

Furthermore, the clinical evidence, primarily derived from large-scale retrospective analyses, shows a promising signal for glaucoma prevention. However, we must interpret these findings with caution, explicitly acknowledging the significant heterogeneity in study design, patient cohorts, and choice of comparator agents that characterizes the current body of literature. This variability limits direct comparisons but also highlights the robustness of the observed protective trends.

## Conclusion

GLP-1RAs represent a potential paradigm shift in ophthalmology, offering a move from treating downstream pathology to modifying underlying disease processes through metabolic, anti-inflammatory, and neuroprotective mechanisms. To realize this potential, a clear clinical translation path is required.

The immediate priority is to address the drug delivery challenge. Investigating local administration through phase 1 safety and pharmacokinetic trials is a critical first step to bypass the BRB and maximize retinal tissue exposure while minimizing systemic side effects. Building on the strong retrospective data, prospective RCTs for glaucoma are the next logical step. These trials must be designed with primary ophthalmic endpoints, such as slowing the rate of RGC loss or preserving visual field, to definitively establish a disease-modifying effect. The ultimate goal is the development of next-generation agents. This could involve engineering GLP-1RAs with enhanced BRB-penetrating properties or creating dual-function molecules that combine metabolic regulation with specific ocular targeting.

Finally, for diseases with conflicting data like AMD, a “risk-benefit” assessment framework is needed. Future research should aim to identify patient subgroups most likely to benefit. For instance, patients with non-exudative AMD and metabolic risk factors may be ideal candidates, while those with nAMD may require caution and close monitoring if systemic GLP-1RA therapy is initiated for metabolic reasons. Addressing these fundamental questions of mechanism, delivery, and rigorous clinical validation will determine whether this remarkably versatile class of drugs can become a new pillar in the ophthalmologist’s armamentarium.

## Data Availability

The datasets analysed during the current study are available from the corresponding author on reasonable request.

## Abbreviations

GLP-1: Glucagon-like peptide-1
DPP-4: Dipeptidyl peptidase-4
NEPs: Neutral endopeptidases
GLP-1RAs: GLP-1 receptor agonists
GLP-1 Rs: GLP-1 receptors
T2DM: Type 2 diabetes mellitus
DR: Diabetic retinopathy
anti-VEGF: Anti-vascular endothelial growth factor
AMD: Age-related macular degeneration
GA: Geographic atrophy
CVOTs: Cardiovascular outcome trials
BRB: Blood-retinal barrier
cAMP: Cyclic adenosine monophosphate
BBB: Blood-brain barrier
AD: Alzheimer’s disease
CVOs: Circumventricular organs
RPE: Retinal pigment epithelial
GPCRs: G protein-coupled receptors
GCL: Ganglion cell layer
GK: Goto-Kakizaki
E4: Exendin-4
ONL: Outer nuclear layer
TXNIP: Thioredoxin-interacting protein
INL: Inner nuclear layer
NFL: Nerve fiber layer
STZ: Streptozotocin
GLAST: Glutamate-aspartate transporter
nAMD: Neovascular age-related macular degeneration
PLGF: Placental growth factor
ICAM-1: Intercellular adhesion molecule-1
FFAs: Free fatty acids
LDs: Lipid droplets
EMT: Epithelial-mesenchymal transition
AMPK: AMP-activated protein kinase
ROS: Reactive oxygen species
MMP: Mitochondrial membrane potential
DAM: Disease-associated microglial
HbA1c: Glycated hemoglobin
CNS: Central nervous system
RGC: Retinal ganglion cell
POAG: Primary open-angle glaucoma
IOP: Intraocular pressure
RCTs: Randomized controlled trials
BMI: Body mass index
NAION: Nonarteritic anterior ischemic optic neuropathy
DED: Dry Eye Disease
DDES: Diabetic dry eye syndrome
